# A dual, catalytic role for the fission yeast Ccr4-Not complex in gene silencing and heterochromatin spreading

**DOI:** 10.1093/genetics/iyad108

**Published:** 2023-06-06

**Authors:** Drice Challal, Alexandra Menant, Can Goksal, Estelle Leroy, Bassem Al-Sady, Mathieu Rougemaille

**Affiliations:** Université Paris-Saclay, CEA, CNRS, Institute for Integrative Biology of the Cell (I2BC), Gif-sur-Yvette 91198, France; Université Paris-Saclay, CEA, CNRS, Institute for Integrative Biology of the Cell (I2BC), Gif-sur-Yvette 91198, France; Department of Microbiology & Immunology, George Williams Hooper Foundation, University of California San Francisco, San Francisco, CA 94143, USA; Université Paris-Saclay, CEA, CNRS, Institute for Integrative Biology of the Cell (I2BC), Gif-sur-Yvette 91198, France; Department of Microbiology & Immunology, George Williams Hooper Foundation, University of California San Francisco, San Francisco, CA 94143, USA; Université Paris-Saclay, CEA, CNRS, Institute for Integrative Biology of the Cell (I2BC), Gif-sur-Yvette 91198, France

**Keywords:** heterochromatic gene silencing, H3K9me3 propagation, Ccr4-Not complex, jumonji protein Epe1

## Abstract

Heterochromatic gene silencing relies on combinatorial control by specific histone modifications, the occurrence of transcription, and/or RNA degradation. Once nucleated, heterochromatin propagates within defined chromosomal regions and is maintained throughout cell divisions to warrant proper genome expression and integrity. In the fission yeast *Schizosaccharomyces pombe*, the Ccr4-Not complex partakes in gene silencing, but its relative contribution to distinct heterochromatin domains and its role in nucleation versus spreading have remained elusive. Here, we unveil major functions for Ccr4-Not in silencing and heterochromatin spreading at the mating type locus and subtelomeres. Mutations of the catalytic subunits Caf1 or Mot2, involved in RNA deadenylation and protein ubiquitinylation, respectively, result in impaired propagation of H3K9me3 and massive accumulation of nucleation-distal heterochromatic transcripts. Both silencing and spreading defects are suppressed upon disruption of the heterochromatin antagonizing factor Epe1. Overall, our results position the Ccr4-Not complex as a critical, dual regulator of heterochromatic gene silencing and spreading.

## Introduction

Eukaryotic genomes organize into gene-rich, euchromatic regions, and transcriptionally repressed heterochromatin domains. The assembly, maintenance, and inheritance of heterochromatin are essential for major biological processes, including gene expression, chromosome segregation, genome stability, and cell fate (Allshire and Madhani 2018; Janssen *et al.* 2018). In the fission yeast *Schizosaccharomyces pombe*, heterochromatin assembles at defined chromosomal regions, including pericentromeric repeats, subtelomeric regions, and the silent mating type locus. These domains are enriched in nucleosomes methylated on histone H3 lysine 9 (H3K9me), a modification catalyzed by the sole Suv39 homolog Clr4 and bound by proteins of the HP1 family (Allshire and Madhani 2018). Such structural components constitute a platform for the recruitment of additional silencing and heterochromatin assembly complexes, including histone-remodeling and modifying enzymes as well as the RNA interference (RNAi) machinery (Grewal 2010; Reyes-Turcu and Grewal 2012; Holoch and Moazed 2015; Martienssen and Moazed 2015). These different effectors tightly cooperate in intricate, often interdependent regulatory pathways to mediate transcriptional (TGS) and co- or *cis* post-transcriptional (C/*cis*-PTGS) gene silencing, which ultimately restrict RNA polymerase II (RNAPII) accessibility and the accumulation of heterochromatic transcripts, respectively. Heterochromatin also engages factors endowed with antisilencing functions, such as the H3K9me antagonizing protein Epe1 (Ayoub *et al.* 2003; Zofall and Grewal 2006; Isaac *et al.* 2007; Trewick *et al.* 2007; Wang *et al.* 2013; Garcia *et al.* 2015), and the dynamic balance between opposite activities is believed to prevent spreading within adjacent euchromatin and maintain heterochromatin throughout cell divisions (Chen *et al.* 2008; Audergon *et al.* 2015; Ragunathan *et al.* 2015; Wang and Moazed 2017; Yu *et al.* 2018).

Despite commonalities in core components of the silencing machinery, the mechanisms that drive heterochromatin-based silencing substantially differ between loci: while RNAi is essential at pericentromeres, it has little impact at telomeres and the mating type locus due to functional redundancy with RNAi-independent mechanisms. The shelterin complex and the C-AMP Response Element-Binding (CREB)-family proteins Atf1/Pcr1 bind to specific *cis*-acting DNA sequences and independently recruit Clr4 to nucleate heterochromatin at these regions (Thon *et al.* 1999; Jia *et al.* 2004; Kanoh *et al.* 2005; Yamada *et al.* 2005; Wang *et al.* 2016; Wang and Moazed 2017; van Emden *et al.* 2019; Nickels *et al.* 2022). The intrinsic capacities of Clr4 to both recognize H3K9me and methylate adjacent nucleosomes define the molecular basis of a “read-write” mechanism by which heterochromatin subsequently propagates throughout entire domains (Zhang *et al.* 2008; Al-Sady *et al.* 2013; Obersriebnig *et al.* 2016; Nickels *et al.* 2021). Heterochromatin spreading from nucleation sites requires the Clr4-mediated transition from H3K9me2 to H3K9me3, which is important for the switch to TGS (Zhang *et al.* 2008; Al-Sady *et al.* 2013; Jih *et al.* 2017). Despite these progresses, our understanding of the mechanisms underlying heterochromatin propagation is still far from being complete.

Beyond RNAi, other RNA processing/degradation machineries contribute to heterochromatic gene silencing (Bühler *et al.* 2007; Wang *et al.* 2008; Reyes-Turcu *et al.* 2011; Zhang *et al.* 2011; Brönner *et al.* 2017). Among these is the conserved, multifunctional Ccr4-Not complex, which contains two catalytic modules: one formed by the RNA deadenylases Ccr4 and Caf1 and another comprising the E3 ubiquitin ligase subunit Mot2/Not4 (Miller and Reese 2012; Wahle and Winkler 2013; Collart 2016; Ukleja *et al.* 2016). Previous studies in *S. pombe* showed that Ccr4-Not mediates the deposition of H3K9me2 at rDNA repeats, subtelomeric regions, and a subset of meiotic genes (Cotobal *et al.* 2015; Sugiyama *et al.* 2016). The complex was also found to act redundantly with the RNAi machinery to target chromatin-bound RNAs for degradation and preserve heterochromatin integrity (Brönner *et al.* 2017). Recently, Ccr4-Not was involved in the control of transcriptional efficiency by limiting the levels of RNAPII-associated heterochromatic transcripts (Monteagudo-Mesas *et al.* 2022). Though, the precise function and contribution of Ccr4-Not to gene silencing, heterochromatin assembly, and spreading remain unclear.

Here, we demonstrate that Ccr4-Not on its own is crucial for heterochromatic gene silencing and spreading. Mechanistically, the deadenylation and ubiquitinylation activities of the complex independently mediate gene repression, maintain heterochromatin integrity and ensure its efficient propagation within the mating type locus and at subtelomeres. We further show that these functions of the complex are antagonized by the jumonji protein Epe1. Together, our findings unveil a dual, catalytic role for Ccr4-Not in gene silencing and heterochromatin spreading, thereby highlighting its biological relevance to preserving genome expression and integrity.

## Materials and methods

### Strains, media, and plasmids

The *S. pombe* strains used in this study are listed in Supplementary Table 1. Strains were generated by transformation following a lithium acetate-based method or by random spore analysis (mating and sporulation on Malt Extract) using a complete medium (YE Broth, Formedium, #PMC0105) supplemented with appropriate antibiotics. Experiments were performed in 1X YE supplemented with 150 mg/L of adenine (ADE, Sigma, #A2786), L-histidine (Sigma, #H8000), L-lysine (Sigma, #L5501), L-leucine (LEU, Sigma, #L8000), and uracil (URA, Sigma, #U750) (YES), while plasmids-containing strains were grown in 1X EMM-LEU-URA minimal medium (EMM-LEU-URA Broth, Formedium, #PMD0810) supplemented with uracil (Sigma, #U750) (EMM-LEU). For silencing assays, cells were grown until midlog phase and plated on both nonselective (YES or EMM-LEU) and selective (YES+5FOA, EMM-LEU+5FOA or YES low ADE) media at an initial OD = 0.2–0.3 followed by 5-fold serial dilutions. Pictures were taken after 3–4 days of growth at 30°C.

The plasmids used for gene cloning/editing are listed in Supplementary Table 2. To construct strains expressing tagged versions of wt or mutant Epe1 from its genomic locus, we first deleted the ORF with a cassette containing the hygromycin resistance marker (hph^R^MX) fused to the herpes simplex virus thymidine kinase-encoding gene (HSV-TK) from the pFA6a-HyTkAX vector (Addgene plasmid #73898; http://n2t.net/addgene:73898; RRID:Addgene_73898) (Amelina *et al.* 2016). The resulting hph^R^ and TK-expressing *epe1Δ* strain were then transformed with a PCR product of interest (i.e. Epe1-TAP or Epe1^H297A^-TAP) carrying homology with the promoter and terminator regions. Positive integrants were selected in the presence of 5-fluoro-2′-desoxyuridine, which counter-selects cells expressing TK and became again hygromycin-sensitive due to cassette pop-out.

### RNA extraction

Total RNAs were extracted from 4 mL of yeast cells at OD = 0.8–1.0. Following centrifugation, cells were washed in water and frozen in liquid nitrogen. Cell pellets were resuspended in 1 volume of TES buffer (10 mM Tris–HCl pH7.5, 5 mM EDTA, 1% SDS) and 1 volume of acid phenol solution pH4.3 (Sigma, #P4682), and incubated for 1 h at 65°C in a thermomixer with shaking. After centrifugation, the aqueous phase was recovered and 1 volume of chloroform (ThermoFisher Scientific, #383760010) was added. Samples were vortexed and centrifugated followed by ethanol precipitation in the presence of 200 mM of lithium acetate. Pellets were resuspended in water and treated with DNAse (Ambion, #AM1906). RNA concentrations were measured with a Nanodrop.

### Reverse transcription and real-time PCR (RT-qPCR)

Two micrograms of DNAse-treated RNAs were denatured at 65°C for 5 min in the presence of strand-specific primers or a mix of random hexamers (ThermoFisher Scientific, #SO142) and oligodT. Reactions were carried out with 100 units of maxima reverse transcriptase (ThermoFisher Scientific, #EP0743) at 50°C for 30 min. The enzyme was then denatured at 85°C for 5 min, and reactions were diluted to a 1:10 ratio. Each experiment included negative controls without reverse transcriptase. Samples were quantified by qPCR with SYBR Green Master Mix and a LightCycler LC480 apparatus (Roche). Measurements were statistically compared using two-tailed *t*-tests with the following *P*-value cutoffs for significance: 0.05 > * > 0.01; 0.01 > ** > 0.001; *** < 0.001. Oligonucleotides used in qPCR reactions are listed in Supplementary Table 3.

### Chromatin immunoprecipitation and real-time PCR (ChIP-qPCR)

Forty- to fifty-milliliter yeast cultures were grown in an appropriate medium until OD = 0.8–1.0. Crosslinking was performed by adding 1% formaldehyde (Sigma, #F8775) for 20 min at 30°C on the shaker and the reaction was quenched with 250 mM glycine (Sigma, #G7126) for 5 min at room temperature. Cells were washed with 1X ice-cold PBS, harvested by centrifugation, and frozen in liquid nitrogen. Pellets were resuspended in 500 µL 150 mM FA buffer (50 mM Hepes-KOH pH7.5, 150 mM NaCl, 1 mM EDTA, 1% Triton X-100, 0.1% Na deoxycholate) in 2 mL screw-cap tubes. One milliliter of ice-cold, acid-washed glass beads (Sigma, #G8772) was added, and cells were lysed by six cycles of 40 s at 6,000 rpm using a FastPrep-24 5G apparatus (MP). Following centrifugation, pellets were resuspended in 150 mM FA buffer and sonicated for six cycles of 30 s at 40% amplitude using a tip probe VibraCell sonifier (Bioblock Scientific). Chromatin extracts were then cleared by centrifugation for 15 min at 14,000 rpm at 4°C. One hundred microliters were set aside as the input control and 200–400 µL aliquots were typically used for immunoprecipitations. One- to two microliters antibodies against total H3 (Abcam, ab1791), H3K9me2 (Abcam, ab1220), and H3K9me3 (Abcam, ab8898) were added to the lysates and samples were incubated at 4°C on a wheel for 2 h or overnight. Four microliters of prewashed Protein-A or Protein-G Dynabeads (Invitrogen, #10001D or #10003D) were next added and samples were incubated for an additional 2 h at 4°C. For immunoprecipitation of TAP or HTP-tagged proteins (Chp1-TAP, Rpb1-HTP, and Epe1-TAP), 4 µL of prewashed rabbit IgG-conjugated M-270 Epoxy Dynabeads (Invitrogen, #14311D) were added and lysates were incubated on a wheel for 1 h at 4°C. Magnetic beads were then washed at room temperature twice with 150 mM FA buffer, twice with 500 mM FA buffer, and once with wash buffer (10 mM Tris–HCl pH8, 0.25 M LiCl, 1 mM EDTA, 0.5% NP-40, 0,5% Na deoxycholate) and once with TE buffer (10 mM Tris–HCl pH8, 1 mM EDTA). DNA was then eluted with 100 µL ChIP Elution buffer (50 mM Tris pH7.5, 10 mM EDTA, 1% SDS) for 20 min at 80°C in a Thermomixer set to 1,400 rpm. Twenty micrograms of proteinase K (Euromedex, #09-911) were added to the input and IP samples for 30 min at 37°C before overnight decrosslinking at 65°C. The following day, 1 µL RNAseA/T1 mix (Thermo Scientific, #EN0551) was added for 30 min at 37°C and DNA was purified using NucleoSpin Gel and PCR Clean-Up columns (Macherey-Nagel, #740609.250) according to the manufacturer's instructions. Samples were quantified by qPCR with SYBR Green Master Mix and a LightCycler LC480 apparatus (Roche). Measurements were statistically compared using two-tailed *t*-tests with the following *P*-value cutoffs for significance: 0.05 > * > 0.01; 0.01 > ** > 0.001; *** < 0.001. Oligonucleotides used in qPCR reactions are listed in Supplementary Table 3.

### Total protein analyses

Total proteins were extracted from cell pellets corresponding to 2–5 ODs, as described in (Simonetti *et al.* 2017). Cell lysis was performed on ice using 0.3 M NaOH and 1% beta-mercaptoethanol before protein precipitation with trichloroacetic acid (TCA) (7% final). Following full-speed centrifugation, pellets were resuspended in HU loading buffer and heat-denaturated at 70°C. Soluble fractions were recovered, and samples were analyzed by standard immunoblotting procedures using 1:3,000 peroxydase-conjugated antiperoxydase (PAP, to detect protein-A-tagged proteins) (Sigma, #P1291, RRID:AB_1079562), 1:3,000 monoclonal anti-FLAG antibody (Sigma, #F3165, RRID:AB_259529), 1:3,000 anti-CDC2 antibody (Abcam, #ab5467, RRID:AB_2074778), 1:1,000 anti-GFP antibody (Roche, #11814460001, RRID: AB_390913) and 1:5,000 goat antimouse IgG-HRP (Santa Cruz Biotechnology, #sc-2005, RRID:AB_631736). Detection was done with SuperSignal West Pico Chemiluminescent Substrate (ThermoFisher Scientific, #34080), ECL Select reagent (GE Healthcare, #RPN2235), and a Vilber Lourmat Fusion Fx7 imager or a ChemiDoc Touch Imaging System (BIORAD).

### Northern blotting

Ten micrograms of RNAs were separated on a 1.2% agarose gel and transferred overnight by capillarity on a nylon membrane (GE Healthcare, #RPN203B). RNAs were then UV-crosslinked to the membrane using a Stratalinker apparatus. A radiolabeled PCR probe was prepared by random priming (GE Healthcare, Megaprime kit) using α-P^32^ dCTP and incubated with the membrane in commercial buffer (Ambion, UltraHyb). Following washes, the membrane was exposed for 24 h and revelation was performed using a Typhoon phosphoimager. Oligonucleotides used to generate the probe are listed in Supplementary Table 3.

### Flow cytometry data collection and normalization for validation

Cells were struck out in YES, grown in liquid YES overnight to saturation, and then diluted 1:15 before further growth in liquid YES up to midlog phase. Flow analysis was performed on an LSR Fortessa X50 (BD Biosciences) and color compensation, analysis, and plotting were performed as described previously (Greenstein *et al.* 2022).

## Results

### The Ccr4-Not complex mediates heterochromatic gene silencing at the mating type locus

**Fig. 1.**
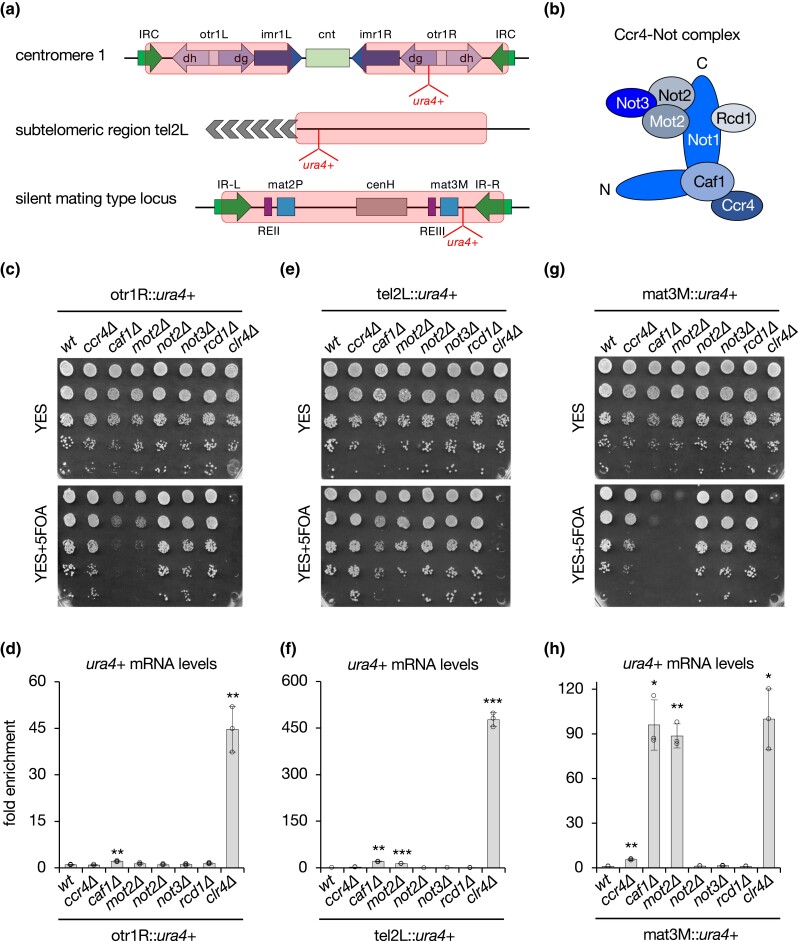
The Ccr4-Not complex mediates heterochromatic gene silencing at the mating type locus. a) Scheme of the different heterochromatin domains with the position of the *ura4*+ reporter gene. Red rectangles define heterochromatin regions. b) Scheme illustrating the different subunits of the core Ccr4-Not complex. c, e and g) 5-fold serial diluted silencing assays using the *ura4*+ reporter gene inserted at pericentromeric repeats (otr1R::*ura4*+), subtelomeric regions (tel2L::*ura4*+), and the mating type locus (mat3M::*ura4*+). Cells of the indicated genotypes were plated on both nonselective (YES) and 5FOA-containing (YES+5FOA) media. Defects in heterochromatic gene silencing result in sensitivity to FOA, which counter-selects cells expressing *ura4*+. d, f and h) RT-qPCR analyses of *ura4*+ transcripts (mean ± SD; *n* = 3; normalized to *act1*+; relative to wt) produced from the different heterochromatic loci in cells of the indicated genetic background. Two-tailed Student's *t*-tests were used to calculate *P*-values. Individual data points are represented by black circles.

To assess the role of the Ccr4-Not complex in heterochromatic gene silencing, we generated strains carrying the *ura4*+ reporter gene inserted at pericentromeric repeats (otr1R::*ura4*+), subtelomeric regions (tel2L::*ura4*+) or the silent mating type cassette (mat3M::*ura4*+) in which nonessential subunits (all but the scaffolding subunit Not1) were deleted (Fig. 1, a and b). Gene silencing was probed by growing cells in the presence of 5FOA, which counter-selects those expressing *ura4*+, and by measuring steady-state *ura4*+ mRNA levels in RT-qPCR assays. A strain lacking the H3K9 methyltransferase Clr4, defective for silencing at all heterochromatic domains, was assayed in parallel as an internal control. As shown in Fig. 1, c and d, we observed a minor defect in pericentromeric silencing in the *caf1Δ* mutant, while *mot2Δ* cells did not accumulate *ura4*+ transcripts despite a partial sensitivity to 5FOA. These two mutants also exhibited a significant increase in *ura4*+ mRNA levels when expressed from subtelomeres, although this was considerably lower than that of cells lacking Clr4 (about 15–20-fold compared to 500-fold) (Fig. 1, e and f). Consistent with these results and previous observations (Brönner *et al.* 2017; Monteagudo-Mesas *et al.* 2022), endogenous dg pericentromeric repeats and subtelomeric *tlh1+* sequences were not or only marginally increased in *caf1Δ* and *mot2Δ* mutants (Supplementary Fig. 1a), arguing against a major contribution of the Ccr4-Not complex per se at these loci.

Strikingly, however, silencing at the mating type locus was completely abolished in the absence of Caf1 and Mot2, similar to *clr4Δ* cells, as revealed by the lack of cell growth on 5FOA-containing medium and the marked accumulation of *ura4*+ transcripts (Fig. 1, g and h). These results were corroborated using strains carrying the *ade6*+ or *gfp*+ reporter genes instead of *ura4*+ (i.e. mat3M::*ade6*+ and mat3M::*gfp*+). Indeed, while pink/red colonies were observed for the wt strain on adenine-limiting medium, reflecting *ade6*+ silencing, *caf1Δ* and *mot2Δ* cells formed white colonies, like the *clr4Δ* mutant, indicative of derepressed *ade6*+ expression (Supplementary Fig. 1b). Steady-state *gfp*+ mRNA levels were also strongly increased in these mutants, which correlated with the accumulation of the Gfp protein itself, supporting that heterochromatic transcripts are efficiently exported and translated in these different genetic backgrounds (Supplementary Fig. 1, c and d). We also detected intermediate phenotypes for the *ccr4Δ* mutant, pointing to a partial contribution of this RNA deadenylase (Fig. 1, g and h; Supplementary Fig. 1, b–d). Moreover, endogenous matMc transcripts strongly accumulated in matP cells lacking Caf1 and Mot2, confirming the requirement for these factors in suppressing the expression of heterochromatic RNAs produced from the mating type locus (Supplementary Fig. 1e). We concluded from these experiments that the RNA deadenylase Caf1 and the E3 ubiquitin ligase Mot2 subunits of the Ccr4-Not complex are major regulators of heterochromatic gene silencing at the mating type locus.

Previous studies, including ours, showed that the Ccr4-Not complex tightly associates with the RNA-binding protein Mmi1 and its partner Erh1 to promote facultative heterochromatin assembly at meiotic genes and rDNA silencing (Cotobal *et al.* 2015; Sugiyama *et al.* 2016; Ukleja *et al.* 2016; Simonetti *et al.* 2017; Xie *et al.* 2019; Hazra *et al.* 2020). We investigated whether these factors also contribute to gene silencing at the mating type locus and found that they were not required (Supplementary Fig. 1, f and g). Hence, Ccr4-Not acts independently of Mmi1 and Erh1.

### The Ccr4-Not complex impacts heterochromatin assembly at the mating type locus

**Fig. 2.**
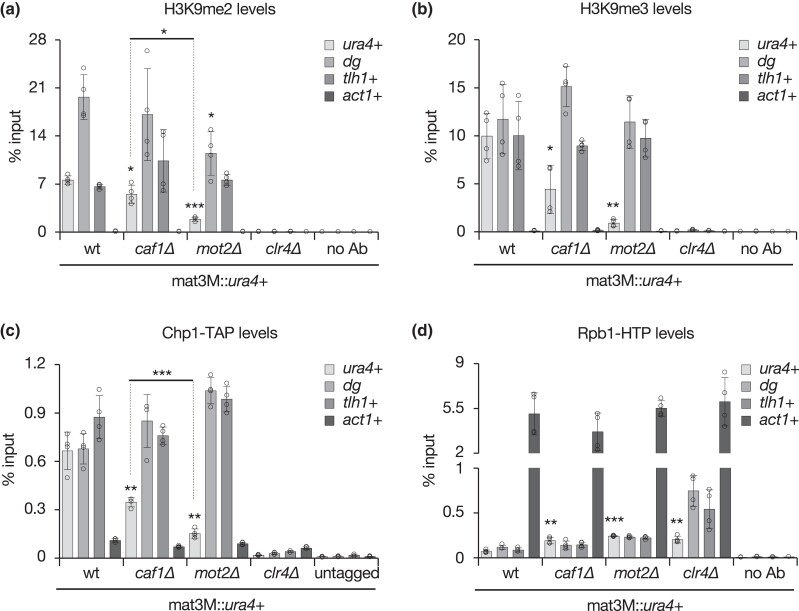
The Ccr4-Not complex impacts heterochromatin assembly at the mating type locus. a and d) ChIP-qPCR analyses (% input; mean ± SD; *n* = 4) of the indicated histone modifications and tagged proteins in mat3M::*ura4*+ strains. Shown are the enrichments of *ura4*+, *dg* pericentromeric repeats, the *tlh1*+ subtelomeric locus, and *act1*+ upon immunoprecipitation with H3K9me2 a), H3K9me3 b) antibodies or rabbit IgG c and d). Immunoprecipitations without antibodies (no Ab) or from untagged strains were performed to determine background levels. In d) “no Ab” values represent the mean of two independent analyses (*n* = 2). Two-tailed Student's *t*-tests were used to calculate *P*-values. Individual data points are represented by black circles.

We next sought to determine whether defective silencing at the mating type locus in Ccr4-Not mutants is accompanied by an alteration in heterochromatin structural components. ChIP experiments revealed that the absence of Caf1 only modestly impacts H3K9me2 at mat3M::*ura4*+, while the *mot2Δ* mutant exhibited a pronounced decrease, albeit lower than that of *clr4Δ* cells (Fig. 2a). We also assessed H3K9me3 levels and observed a further reduction in both *caf1Δ* and *mot2Δ* cells when compared to the wt strain (Fig. 2b). Importantly, these defects were restricted to the mat3M locus, as H3K9me2/3 levels at pericentromeric dg repeat and subtelomeric *tlh1*+ sequences remained similar to those detected in wt cells, with the exception of a small H3K9me2 decrease at dg in the absence of Mot2 (Fig. 2, a and b). Total H3 levels were similar in all strains (Supplementary Fig. 2a), excluding an indirect effect linked to nucleosome instability. Since H3K9me2/3 constitutes a docking site for proteins of the HP1 family, we next analyzed the recruitment of the chromodomain-containing RNA-Induced Transcriptional Silencing complex (RITS) subunit Chp1 involved in RNAi (Noma *et al.* 2004; Verdel *et al.* 2004). Following a similar trend, Chp1 occupancy was reduced specifically at mat3M::*ura4*+ in Ccr4-Not mutants (Fig. 2c). Together, our results unveil a role for Caf1 and Mot2 in heterochromatin assembly, which correlates with their requirement for gene silencing at the mating type locus (Fig. 1, g and h). They further suggest that both factors operate differentially, Mot2 being more critical for heterochromatin integrity.

Clr4-mediated heterochromatin assembly at centromeres and telomeres is essential to limit RNAPII accessibility and hence trigger efficient TGS, whereas its impact on the transcription machinery at the mating type locus is moderate, reflecting a predominance of C/*cis*-PTGS (Bühler *et al.* 2007). In agreement with these notions, we found that RNAPII strongly accumulated at dg and *tlh1*+ sequences but only modestly at mat3M::*ura4*+ in the absence of Clr4 (Fig. 2d). In *caf1Δ* and *mot2Δ* mutants, RNAPII levels increased similarly at mat3M but only marginally at pericentromeric or subtelomeric loci when compared to *clr4Δ* cells (Fig. 2d), consistent with the relative impact of Ccr4-Not in silencing/heterochromatin formation at these different regions. We also assessed whether Caf1 and Mot2 themselves are recruited to the mating type locus but failed to detect a significant enrichment, as opposed to the RITS component Chp1 (Supplementary Fig. 2b). However, Caf1 binds to chromatin-associated RNAs (Brönner *et al.* 2017), including at the mating type locus, and the Ccr4 subunit physically interacts with Chp1 (Cotobal *et al.* 2015), which together supports a direct role for Ccr4-Not in heterochromatic gene silencing.

### The Ccr4-Not subunits Caf1 and Mot2 regulate heterochromatin spreading

**Fig. 3.**
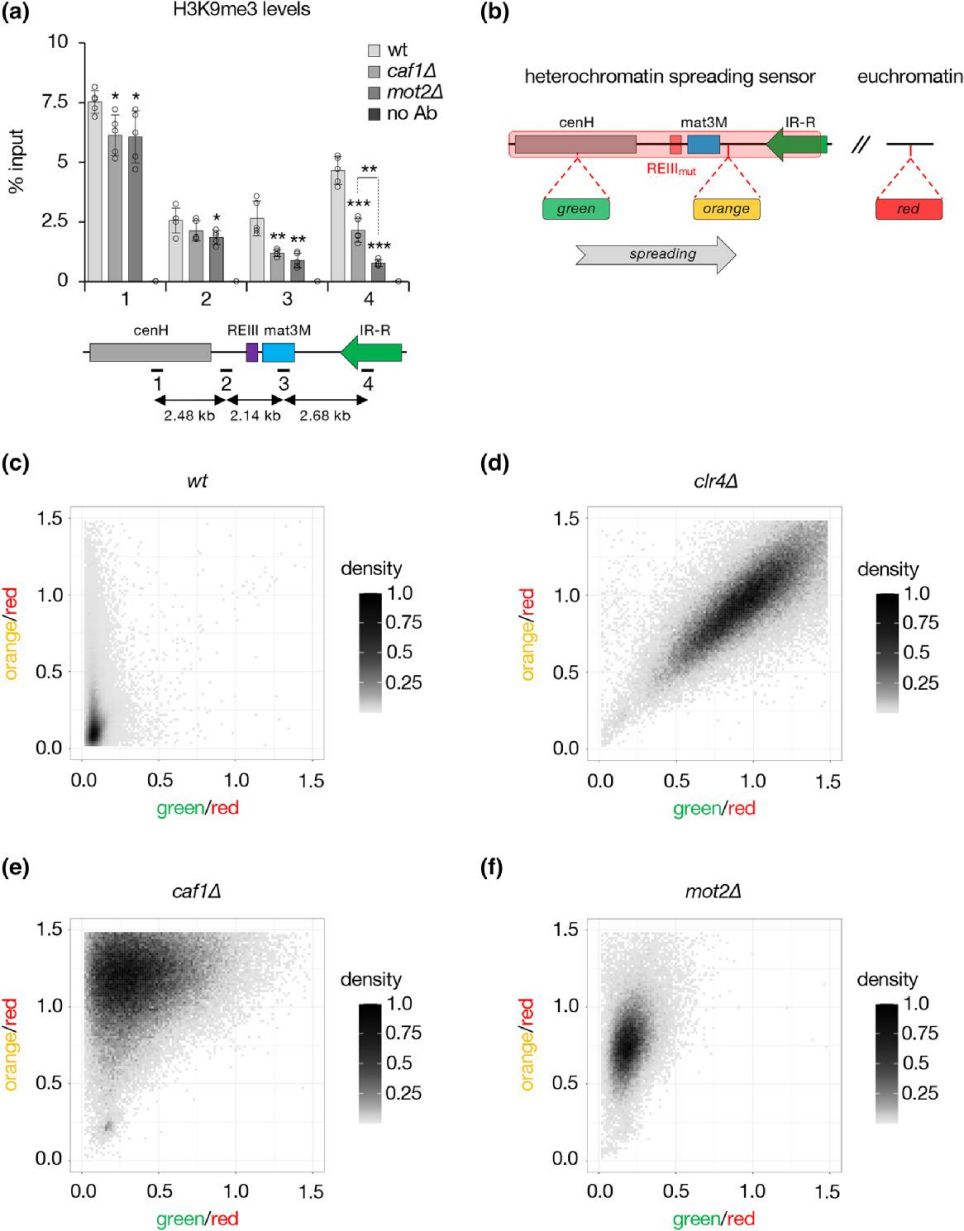
The Ccr4-Not subunits Caf1 and Mot2 regulate heterochromatin spreading. a) H3K9me3 ChIP-qPCR analyses (% input; mean ± SD; *n* = 5) in cells of the indicated genetic backgrounds. Numbers correspond to the different primer pairs used in qPCR reactions and whose localization is indicated on the scheme below the graph. Immunoprecipitations without antibodies (no Ab) were performed to determine background levels. Two-tailed Student's *t*-tests were used to calculate *P*-values. Individual data points are represented by black circles. b) Scheme depicting the heterochromatin spreading sensor with the relative positions of the “green,” “orange,” and “red” reporters. c–f) Two-dimensional-density square bin plots showing the red-normalized green and orange fluorescence for wt c), *clr4Δ* d), *caf1Δ* e), and *mot2Δ* f) REIII_mut_ cells grown at 32°C. A density bar represents the fraction of the most dense bin. One representative isolate is shown for each background.

The transition from H3K9me2 to H3K9me3 contributes to heterochromatin spreading from nucleation sites (Al-Sady *et al.* 2013; Jih *et al.* 2017). Our findings that *caf1Δ* and *mot2Δ* cells predominantly affect H3K9me3 at mat3M::*ura4*+ prompted us to examine their potential impact on heterochromatin spreading. Intriguingly, ChIP experiments revealed a gradual decrease of H3K9me3 in Ccr4-Not mutants relative to the wt strain, from the cenH nucleation center up to inverted repeats at the right border (IR-R) of the mating type locus (Fig. 3a). Consistent with the above results, the effect was also more prominent in the absence of Mot2.

To substantiate these findings, we used our previously described fluorescent reporter-based, single-cell spreading sensor assay (Greenstein *et al.* 2018, 2020), whereby three different fluorescent protein-coding genes allow quantitative measurements of gene expression at nucleation-proximal (“green”) and -distal (“orange”) heterochromatin sites, as well as at a euchromatic locus (“red”) for signal normalization purposes (Fig. 3b). Because heterochromatin at the mating type locus can nucleate from two distinct regulatory DNA elements (i.e. cenH and REIII), we analyzed strains in which the Atf1/Pcr1-binding sites within REIII are mutated (REIII_mut_), thereby allowing to record heterochromatin spreading (“orange”) from the sole cenH nucleation center (“green”) (Fig. 3b).

Flow cytometry analyses revealed that wt REIII_mut_ cells successfully nucleated heterochromatin (“green”^OFF^), which efficiently spread to the distal reporter (“orange”^OFF^), as indicated by the strong enrichment of cell populations in the bottom left part of the 2D density square bin plot (Fig. 3c). A minor fraction of cells displayed some “orange” signal though, reflecting a certain degree of stochasticity in the spreading process (Greenstein *et al.* 2018, 2022). Upon complete loss of heterochromatin and silencing (i.e. in *clr4Δ* cells), populations concentrated along the diagonal, in the upper right part of the plot, consistent with both reporters being fully derepressed (“green”^ON^ and ^“^orange”^ON^) (Fig. 3d). Remarkably, Ccr4-Not mutants exhibited radically different patterns, with a strong bias for “orange” versus “green” fluorescence overall (Fig. 3, e and f; Supplementary Fig. 3, a–d). In the absence of Caf1, the majority of cells distributed within a broad range of high “orange” and intermediate “green” signals (Fig. 3e; Supplementary Fig. 3, a and b). This indicates that the deadenylase strongly suppresses the expression of the nucleation-distal reporter while only partially contributing to the silencing of the proximal locus. Of note, we reproducibly observed a subpopulation in the bottom left part of the plot, indicating that some cells maintained both reporters in the repressed state. Cells lacking Mot2 showed instead a narrow, vertical distribution of fluorescence, covering a large range of high “orange” intensities associated with low “green” signals (Fig. 3f; Supplementary Fig. 3, c and d). Such a pattern is reminiscent of what was previously observed for *bona fide* regulators of heterochromatin spreading (Greenstein *et al.* 2022). Consistent with these results, cenH proximal-transcripts were only partially increased in Ccr4-Not mutants when compared to *clr4Δ* cells (Supplementary Fig. 3e), in striking contrast to distal matMc RNAs (Supplementary Fig. 1e).

Because the pericentromeric and subtelomeric reporter strains used in this study (i.e. otr1R::*ura4*+ and tel2L::*ura4*+; Fig. 1, c–f) carry the *ura4*+ gene in proximity to nucleation sites (i.e. dg and *tlh1*+ sequences, respectively), we next investigated heterochromatin propagation at these regions by ChIP. While H3K9me3 distribution remained similar to wt cells at pericentromeres (Supplementary Fig. 4a), *caf1Δ* and *mot2Δ* mutants exhibited lower levels away from the subtelomeric *tlh1*+ gene (Supplementary Fig. 4b), consistent with former observations for H3K9me2 (Cotobal *et al.* 2015; Brönner *et al.* 2017). Expression analyses further revealed that Caf1 suppresses distal subtelomeric transcripts similar to Clr4 (Supplementary Fig. 4c, see primer pairs 4 and 5), while its role in the repression of proximal loci was in comparison much less pronounced (Supplementary Fig. 4c, see primer pairs 1 and 2). The contribution of Mot2 followed a similar tendency, although the most distal genes were instead downregulated in its absence, likely due to additional effects impairing the expression of these loci (Supplementary Fig. 4c).

Overall, our data establish critical, yet different roles for Caf1 and Mot2 in the propagation of H3K9me3 and the repression of nucleation-distal transcripts, pointing to key contributions of Ccr4-Not to heterochromatin spreading at the mating type locus and subtelomeres.

### Importance of Caf1 and Mot2 catalytic activities in gene silencing and heterochromatin spreading

**Fig. 4.**
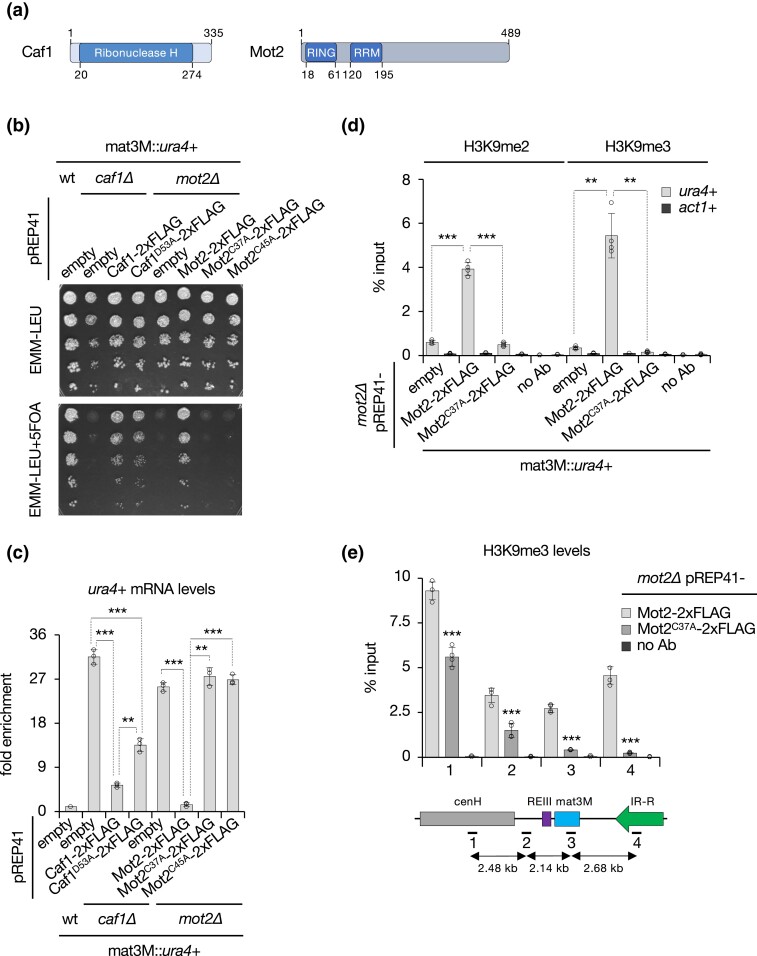
Importance of Caf1 and Mot2 catalytic activities in gene silencing and heterochromatin spreading. a) Domain organization of Caf1 and Mot2 proteins. b) 5-fold serial diluted silencing assay using the *ura4*+ reporter gene inserted at the mating type locus (mat3M::*ura4*+). Cells of the indicated genotypes were plated on both nonselective (EMM-LEU) and 5FOA-containing (EMM-LEU+5FOA) media. c) RT-qPCR analyses of *ura4*+ transcripts (mean ± SD; *n* = 3; normalized to *act1*+; relative to wt pREP41) in cells of the indicated genetic backgrounds. d and e) H3K9me2 and H3K9me3 ChIP-qPCR analyses (% input; mean ± SD; *n* = 4 or 3) in cells of the indicated genetic backgrounds. Immunoprecipitations without antibodies (no Ab) were performed to determine background levels. In e) numbers correspond to the different primer pairs used in qPCR reactions and whose localization is indicated on the scheme below the graph. c–e) Two-tailed Student's *t*-tests were used to calculate *P*-values. Individual data points are represented by black circles.

To determine the mechanisms by which Caf1 and Mot2 mediate heterochromatic gene silencing and spreading, we assessed the impact of mutants defective for their RNA deadenylation and E3 ubiquitin ligase activities, which are carried by Ribonuclease H superfamily and RING-type domains respectively (Fig. 4a).

We first expressed a plasmid-borne, 2xFLAG-tagged version of Caf1^D53A^ mutant, previously shown to impair RNA deadenylation (Stowell *et al.* 2016), in *caf1Δ* mat3M::*ura4*+ cells (Supplementary Fig. 5a). Although we observed only a mild growth defect in the presence of 5FOA (Fig. 4b), *ura4*+ mRNA levels were significantly increased, yet not as pronounced as *caf1*+ deletion (Fig. 4c). This likely reflects the contribution of the second deadenylase Ccr4 (Fig. 1h; Supplementary Fig. 1, b–d) (Brönner *et al.* 2017), which is physically tethered to the complex by Caf1 (Basquin *et al.* 2012) and hence does not exert its function in *caf1Δ* cells. Of note, a plasmid-borne, 2xFLAG-tagged wild type Caf1, did not allow us to fully restored growth on 5FOA nor low levels of *ura4*+ transcripts in *caf1Δ* cells (Fig. 4, b and c), indicating that ectopic expression of Caf1 also partially inhibits silencing. Overall, these results indicate that the RNA deadenylation activity of Caf1 partially contributes to heterochromatic gene silencing.

Next, we assessed the requirement for Mot2 catalytic activity following the same strategy. Expression of a plasmid-borne Mot2^RINGΔ^ mutant in otherwise *mot2Δ* cells completely abolished *ura4*+ silencing, as determined by the lack of growth in the presence of 5FOA and RT-qPCR analyses (Supplementary Fig. 5, b and c). We further studied mutants in which key cysteine residues in the RING domain were substituted by alanine (Mot2^C37A^, Mot2^C45A^) (Simonetti *et al.* 2017) and similarly observed the absence of growth on 5FOA-containing medium as well as a strong accumulation of *ura4*+ mRNAs, akin to the *mot2Δ* strain (Fig. 4, b and c). Importantly, these phenotypes did not result from a lowered expression of the mutant proteins (Supplementary Fig. 5d). Since the absence of Mot2 strongly impairs heterochromatin assembly at the mating type locus (Fig. 2, a and b), we also investigated the impact of Mot2^C37A^ on H3K9me at this region. ChIP analyses revealed a marked decrease in both H3K9me2 and H3K9me3 levels at mat3M::*ura4*+, similar to *mot2Δ* cells (Fig. 4d). We further assessed the distribution of H3K9me3 from the cenH nucleation center to inverted repeats and reproducibly observed a progressive decrease (Fig. 4e), strongly suggesting a defect in the spreading process. Hence, we concluded that the E3 ubiquitin ligase activity of Mot2 has a major role in gene silencing, heterochromatin assembly, and spreading.

### The antisilencing factor Epe1 opposes Ccr4-Not in gene silencing and heterochromatin spreading

**Fig. 5.**
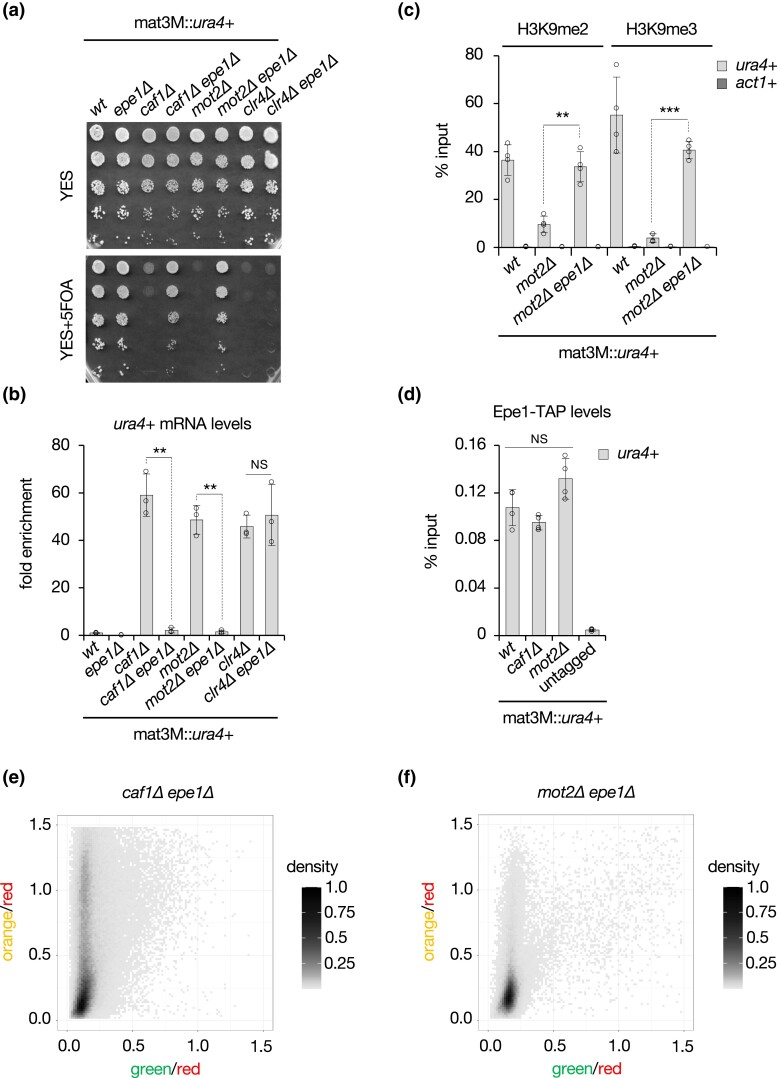
The antisilencing factor Epe1 opposes Ccr4-Not in gene silencing and heterochromatin spreading. a) 5-fold serial diluted silencing assays using the *ura4*+ reporter gene inserted at the mating type locus (mat3M::*ura4*+). Cells of the indicated genotypes were plated on both nonselective (YES) and 5FOA-containing (YES+5FOA) media. b) RT-qPCR analyses of *ura4*+ transcripts (mean ± SD; *n* = 3; normalized to *act1*+; relative to wt) in cells of the indicated genetic backgrounds. c) H3K9me2 and H3K9me3 ChIP-qPCR analyses (% input; mean ± SD; *n* = 4) in cells of the indicated genetic backgrounds. d) Epe1-TAP ChIP-qPCR analyses (% input; mean ± SD; *n* = 4) in cells of the indicated genetic backgrounds. An untagged strain was used as a negative control. b–d) Two-tailed Student's *t*-tests were used to calculate *P*-values. Individual data points are represented by black circles. e and f) Two-dimensional-density squarebin plots showing the red-normalized green and orange fluorescence for *caf1Δ epe1Δ* e) and *mot2Δ epe1Δ* f) REIII_mut_ cells grown at 32°C. A density bar represents the fraction of the most dense bin. One representative isolate is shown for each background.

Previous studies showed that deletion of the JmjC domain-containing protein Epe1 suppresses silencing defects observed in mutants of the heterochromatin machinery (Ayoub *et al.* 2003; Zofall and Grewal 2006; Braun *et al.* 2011; Aygün *et al.* 2013; Barrales *et al.* 2016; Murawska *et al.* 2021). To determine whether Epe1 also opposes the function of Ccr4-Not, we constructed *caf1Δ epe1Δ* and *mot2Δ epe1Δ* double mutants and assessed their ability to restore heterochromatic gene silencing at the mating type locus. Interestingly, silencing defects in the absence of Caf1 and Mot2 were completely suppressed upon *epe1*+ deletion (Fig. 5a). This was in marked contrast to *clr4Δ* cells, consistent with the notion that H3K9me is required for Epe1 recruitment. RT-qPCR assays and Northern blotting further confirmed that the absence of Epe1 restricts the accumulation of *ura4*+ mRNAs in *caf1Δ* and *mot2Δ*, but not *clr4Δ* mutants (Fig. 5b; Supplementary Fig. 6a). Likewise, defects in H3K9me2 and H3K9me3 in cells lacking Mot2 were alleviated upon loss of Epe1 (Fig. 5c), further indicating that the latter mediates the heterochromatin assembly defects. These results also suggest that *caf1Δ* and *mot2Δ* cells maintain sufficient levels of H3K9me to ensure Epe1 recruitment. Indeed, ChIP experiments revealed that the protein was similarly enriched at mat3M::*ura4*+ when compared to the wt strain (Fig. 5d), and Western blot analyses further excluded an alteration in Epe1 protein levels in the mutants (Supplementary Fig. 6b).

To determine the contribution of Epe1 to the heterochromatin spreading defects observed in Ccr4-Not mutants, we next performed flow cytometry analyses in the *caf1Δ epe1Δ* and *mot2Δ epe1Δ* strains as described above. In both cases, cell populations with a high spreading marker (“orange”) fluorescence was strongly diminished compared to single mutants, yet not fully to wt levels (Fig. 5, e and f). In particular, *caf1Δ epe1Δ* populations still distributed over a broad range of “orange” signals, while “green” fluorescence was comparable to what was observed in the wt. *mot2Δ epe1Δ* cells also displayed a pronounced reduction in “orange” and maintained low levels of “green.” We concluded from these experiments that the loss of Epe1 strongly limits the extent and penetrance of heterochromatin spreading defects in Ccr4-Not mutants, akin to its effects on gene repression and heterochromatin formation.

To get additional insights into the mechanism by which it antagonizes Ccr4-Not, we subsequently generated an Epe1 mutant in the JmjC domain (i.e. Epe1^H297A^) that was inserted at its endogenous locus and expressed from its own promoter (Supplementary Fig. 7a). Combining this allele to the deletion of *caf1*+ or *mot2*+ fully restored cell growth in the presence of 5FOA and suppressed the accumulation of *ura4*+ mRNAs produced from the mat3M locus (Supplementary Fig. 7, b and c). We verified by Western blotting that the mutant protein was similarly expressed to the wt version in the different genetic backgrounds (Supplementary Fig. 7d). Consistent with former work (Raiymbek *et al.* 2020), ChIP experiments further indicated that Epe1^H297A^ fails to be efficiently recruited to heterochromatin (i.e. mat3M::*ura4*+) (Supplementary Fig. 7e), providing a rationale for the suppression of silencing defects in *caf1Δ* and *mot2Δ* cells. Thus, the loss of heterochromatic gene silencing in mutants of the Ccr4-Not complex strictly depends on the integrity of the Epe1 JmjC domain.

## Discussion

In this study, we demonstrate that the Caf1 and Mot2 subunits of Ccr4-Not are two major catalytic effectors of heterochromatic gene silencing and spreading. Our findings not only go beyond former observations by establishing an essential role for the complex in silencing at the mating type locus but also add another layer to its regulatory control, i.e. the implication of its catalytic components in heterochromatin propagation.

The nuclease module of the Ccr4-Not complex comprises the Caf1 and Ccr4 deadenylases, the former anchoring the latter to the Not1 scaffolding subunit (Basquin *et al.* 2012). Consistent with this and previous analyses implicating the nuclease activity of both enzymes in RNA degradation and heterochromatic gene silencing (Stowell *et al.* 2016; Brönner *et al.* 2017), we found that (1) catalytically inactive Caf1 elicits weaker defects than its deletion and, (2) Ccr4 partially contributes to the gene silencing activity. Caf1 and Ccr4 may thus act in concert with polyadenylated, heterochromatic RNAs, although it remains unclear whether they target common or individual transcripts. Both enzymes may further promote the recruitment of additional RNA degradation factors important for silencing activities, as previously suggested (Brönner *et al.* 2017). The fact that the heterologous mat3M::*gfp*+ reporter is derepressed in the absence of Caf1 also makes it unlikely that defined factor(s) tether the complex to heterochromatic transcripts in a sequence-dependent manner. It is however possible that poly(A)-binding proteins and/or the Mot2 subunit itself, which carries an RNA Recognition Motif, may recruit Ccr4-Not to promote deadenylation (Kerr *et al.* 2011; Webster *et al.* 2018; Yi *et al.* 2018). Alternatively, the complex may directly associate with the transcription machinery, as illustrated by its interaction in budding yeast with RNAPII and the histone chaperone Spt6 (Kruk *et al.* 2011; Dronamraju *et al.* 2018), both of which also contribute to heterochromatic gene silencing in fission yeast (Djupedal *et al.* 2005; Kato *et al.* 2005, 2013; Kiely *et al.* 2011). Regardless of the precise modalities, the recruitment of Ccr4-Not deadenylases is essential to clear RNAs from chromatin, thereby maintaining heterochromatin integrity (this study; Brönner *et al.* 2017).

Our work also establishes a potent, catalytic role for the E3 ubiquitin ligase Mot2 in gene silencing and heterochromatin assembly. A parsimonious model predicts that Mot2 may target an antisilencing factor for ubiquitinylation-dependent proteasomal degradation, as previously described for the E3 ligase Ddb1 that promotes Epe1 turnover and restricts its accumulation within heterochromatin domains (Braun *et al.* 2011). However, this factor is unlikely to be such a substrate given its similar abundance and occupancy in wt and *mot2Δ* cells. Mot2 could instead target other functionally related factors, including the bromodomain-containing protein Bdf2 and the histone acetyltransferase Mst2 (Wang *et al.* 2013, 2015; Flury *et al.* 2017), thereby shielding heterochromatin from invading antisilencing activities. Other scenarios include the modification of RNAPII itself, perhaps to stimulate elongation on heterochromatin templates, akin to situations where RNAPII transiently pauses or encounters transcriptional blocks (Kruk *et al.* 2011; Babbarwal *et al.* 2014; Dutta *et al.* 2015; Jiang *et al.* 2019). Persistence of the transcription machinery, as suggested by our analyses, and/or the accumulation of transcripts in the vicinity of the DNA template may in turn alter heterochromatin structure (Brönner *et al.* 2017). Interestingly, Ccr4-Not components, including Mot2 and Caf1, were recently proposed to regulate transcriptional efficiency at heterochromatic loci by limiting the levels of RNAPII-associated transcripts (Monteagudo-Mesas *et al.* 2022). The causal relationship between this process and silencing efficiency (i.e. steady-state RNA levels) remains however opaque.

Another critical aspect of our findings is the requirement for both Caf1 and Mot2 in heterochromatin spreading. This is supported by several lines of evidence in mutant cells: (1) H3K9me3 is further reduced than H3K9me2 at the mat3M locus, consistent with a defect in the transition between these two heterochromatin states, (2) propagation of H3K9me3 from nucleation centers is impaired at the mating type locus and subtelomeres, (3) nucleation-distal reporters/genes are further derepressed than proximal loci, and (4) the absence of Epe1, which antagonizes heterochromatin spreading, suppresses the observed silencing and spreading defects. Further supporting a role for Ccr4-Not in heterochromatin spreading, the Ccr4 and Rcd1 subunits were also recently identified in our screen for modulators of spreading at the mating type locus (Greenstein *et al.* 2022). From a mechanistic perspective, however, the exact role of the complex still needs to be elucidated. It is possible that the elimination of RNAPII/chromatin-associated transcripts by Ccr4 and Caf1 facilitates the transition from transcriptionally permissive H3K9me2 to H3K9me3 by Clr4 (Brönner *et al.* 2017; Jih *et al.* 2017; Monteagudo-Mesas *et al.* 2022), thereby ensuring efficient spreading. Their minor contribution to RNA degradation at nucleation centers (i.e. cenH and *tlh1*+) underlies functional redundancy with RNAi (Brönner *et al.* 2017), yet deadenylases may take over at distal loci due to the lack of siRNA templates. As for Mot2, the enzyme could, beyond the scenarios evoked above, directly control the activity of spreading regulators (Murawska *et al.* 2021; Greenstein *et al.* 2022), possibly through nonproteolytic ubiquitinylation. Future studies aimed at identifying the substrate of Mot2 will be instrumental to understand the molecular basis of its involvement in heterochromatin spreading.

Despite reduced H3K9me2/3 levels, *caf1Δ* and *mot2Δ* cells maintain Epe1 occupancy at the mat3M locus, suggesting that the balance between pro- and antisilencing activities is altered. Perhaps Epe1 facilitates heterochromatin transcription and/or histone turnover in these genetic contexts (Zofall and Grewal 2006; Aygün *et al.* 2013; Bao *et al.* 2019), thereby accounting for the observed mutant phenotypes. Our data showing that the protein opposes Ccr4-Not in a JmjC domain-dependent manner might also indicate that failure to antagonize H3K9me allows restoring silencing and spreading activities. However, the JmjC domain of Epe1 does not support H3K9 demethylase activity in vitro but is instead crucial for a robust association with Swi6/HP1 in vivo (Tsukada *et al.* 2006; Raiymbek *et al.* 2020). It is therefore conceivable that the disrupted interaction between Epe1^H297A^ and Swi6 is the main reason why silencing is re-established in Ccr4-Not mutants. Indeed, Epe1^H297A^ is not properly recruited to HP1-coated heterochromatin (this study; Raiymbek *et al.* 2020), which may restrict antisilencing activities and hence ensure efficient H3K9me3 maintenance and spreading.

Heterochromatin domains are spatially segregated at the nuclear periphery within distinct subcompartments (Holla *et al.* 2020; Iglesias *et al.* 2020; Oh *et al.* 2022). The nuclear envelope protein Amo1 was shown to anchor the mating type locus and to stably associate with the Ccr4-Not complex (Holla *et al.* 2020), raising the possibility that the latter may also regulate perinuclear sequestration of this heterochromatin domain. Whether additional peripheral proteins may similarly interact with Ccr4-Not to co-regulate telomere positioning remains an open question. Beyond these considerations, Amo1 cooperates with the rixosome and the histone chaperone FACT to mediate RNA degradation and suppress histone turnover for proper silencing and epigenetic inheritance of heterochromatin (Charlton *et al.* 2020; Holla *et al.* 2020; Shipkovenska *et al.* 2020). Intriguingly, Ccr4 was identified in a screen for factors involved in heterochromatin inheritance and Caf1 was found to act in parallel to the rixosome for the degradation of heterochromatic RNAs (Shipkovenska *et al.* 2020), pointing to a tight relationship between these machineries. Whether Ccr4-Not also partakes in histone turnover and epigenetic inheritance of heterochromatin are fascinating possibilities requiring further investigation.

In conclusion, we demonstrate a fundamental role for the highly conserved Ccr4-Not complex in gene silencing, heterochromatin assembly, and spreading, which implicates its Caf1 and Mot2 subunits. Our findings open new perspectives to dissect the deadenylation- and ubiquitinylation-dependent mechanisms involved and assess the functional and biological relevance of such activities. In *S. pombe*, heterochromatin spreading by Ccr4-Not may be an important regulatory component of mating type switching, a process that determines the mating type of a haploid cell (either P or M) through regulated recombination between the expressed *mat1*+ gene and one of the two adjacent donor cassettes, mat2P or mat3M, embedded in heterochromatin (Thon *et al.* 2019; Arcangioli and Gangloff 2023). Whether the lack of Caf1 or Mot2 introduces biases in donor selection, akin to mutants of various hetero- and euchromatic factors (Maki *et al.* 2018; Esquivel-Chavez *et al.* 2022), requires further analyses. Our study may also be relevant to human disease-related models. The cooperation between human CCR4-NOT and the H3K9me3-functionally linked Human Silencing Hub (HUSH) complex in Human Immunodeficiency Virus (HIV) repression provides a meaningful example in this respect (Tchasovnikarova *et al.* 2015; Matkovic *et al.* 2022).

## Supplementary Material

iyad108_Supplementary_Data

## Data Availability

Strains and plasmids are available upon request. The authors affirm that all data necessary for confirming the conclusions of the article are present within the article, figures, and tables. All the raw data allowing to generate the final results presented here are available in a separate excel file “Supplementary Data 1.” The R script and primary fcs files used to establish flow cytometry data have been deposited in Zenodo (doi: 10.5281/zenodo.7611852). Supplemental material available at GENETICS online.
