# Supplementary material for: A dual, catalytic role for the fission yeast Ccr4-Not complex in gene silencing and heterochromatin spreading
Source: Genetics. 2023 Jun 6;224(4):iyad108. doi: 10.1093/genetics/iyad108 (PMC10411572; doi:10.1093/genetics/iyad108)
Supplement: iyad108_Supplementary_Data [file iyad108_supplementary_data.zip › Supplementary_Figure_6_GENETICS-2023-306219.pdf]

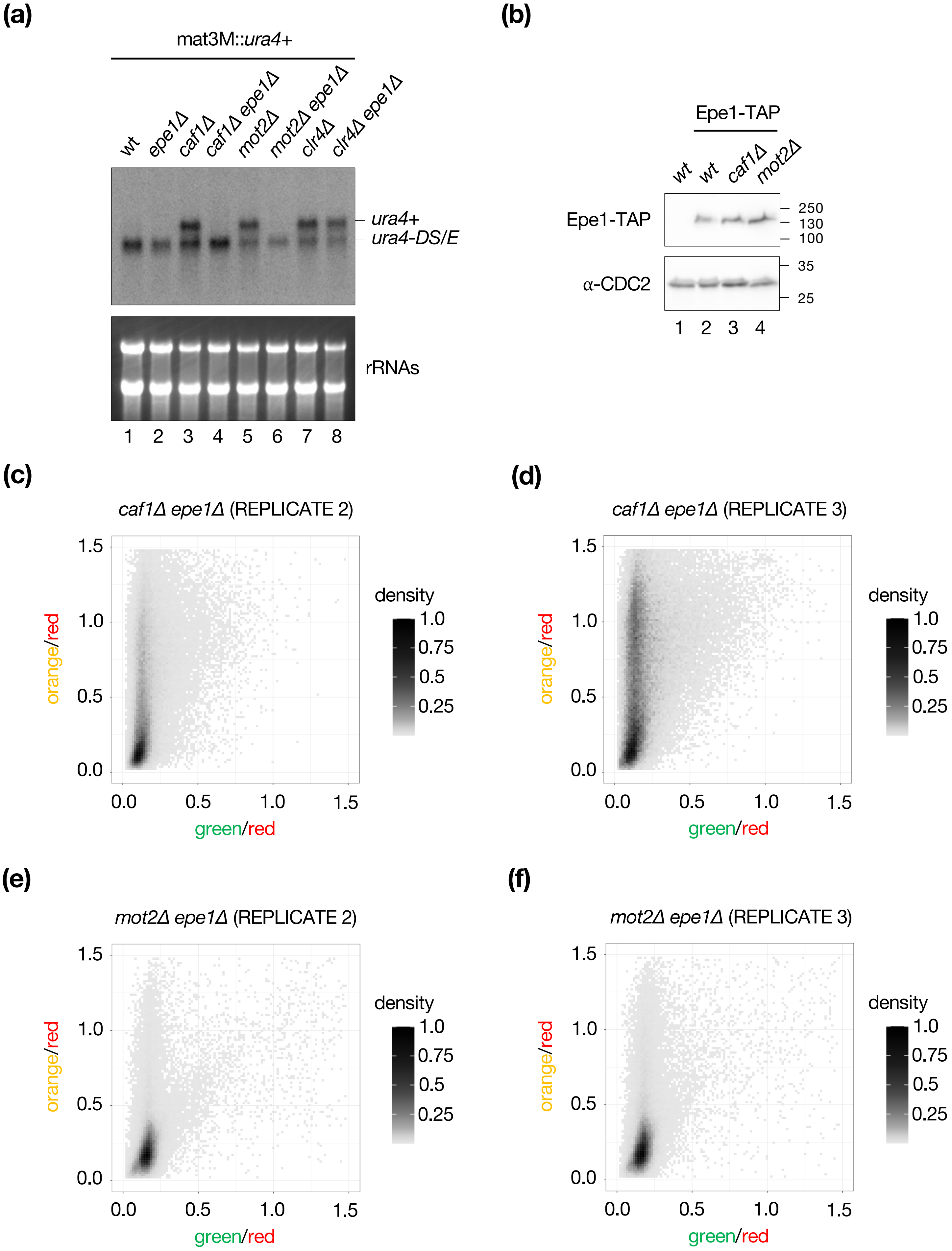

Supplementary Fig. 6. The anti-silencing factor Epe1 opposes Ccr4-Not in gene silencing and heterochromatin spreading.
