# Supplementary material for: A dual, catalytic role for the fission yeast Ccr4-Not complex in gene silencing and heterochromatin spreading
Source: Genetics. 2023 Jun 6;224(4):iyad108. doi: 10.1093/genetics/iyad108 (PMC10411572; doi:10.1093/genetics/iyad108)
Supplement: iyad108_Supplementary_Data [file iyad108_supplementary_data.zip › Supplementary_Figure_Legends_GENETICS-2023-306219.pdf]

Supplementary Figure Legends

Supplementary Fig. 1 (related to Fig. 1). The Ccr4-Not complex mediates heterochromatic gene silencing at the mating type locus. a), c), e), g) RT-qPCR analyses of *dg*, *tlh1+* (**a**), *gfp+* (**c**), *matMc* (**e.**) and *ura4+* (**g**) transcripts (mean±SD; n=3; normalized to *act1+*; relative to wt) in cells of the indicated genetic backgrounds. Two-tailed Student's *t*-tests were used to calculate *p*-values. Individual data points are represented by black circles. **b), f)** 5-fold serial diluted silencing assays using the *ade6+* (**b**) or *ura4+* (**f**) reporter genes inserted at the mating type locus (mat3M::*ade6+*; mat3M::*ura4+*). Cells of the indicated genotypes were plated on both non-selective (YES) and selective (YES low ADE or YES+5FOA) media. Defects in heterochromatic gene silencing result in the formation of white colonies (mat3M::*ade6+*) (**b**) or sensitivity to 5FOA (mat3M::*ura4+*) (**f**). In **f)**, *mmi1*Δ cells were also deleted for *mei4+*, since the absence of Mmi1 leads to major growth defects due to the ectopic expression of the meiosis-specific transcription factor Mei4. The mutants of interest were constructed in a *mei4*Δ background for direct comparison. **d)** Western blot showing total Gfp levels in mat3M::*gfp+* cells of the indicated genetic backgrounds. Anti-CDC2 antibody was used as loading control.

Supplementary Fig. 2 (related to Fig. 2). The Ccr4-Not complex impacts heterochromatin assembly at the mating type locus. a-b) ChIP-qPCR analyses (% input; mean±SD; n=4 or 3) of histone H3 (**a**) and TAP-tagged proteins (**b**) in cells of the indicated genetic backgrounds. Immunoprecipitations without antibodies (no Ab) or from untagged strains were performed to determine background levels. Shown are the enrichments of *ura4+*, *dg* repeats, *tlh1+* and *act1+* upon immunoprecipitation with H3 antibody (**a**) or rabbit IgG (**b**). Individual data points are represented by black circles.

Supplementary Fig. 3 (related to Fig. 3). The Ccr4-Not subunits Caf1 and Mot2 regulate heterochromatin spreading at the mating type locus. a-d) Two-dimensional-density squarebin plots showing the red-normalized green and orange fluorescence for *caf1Δ* (**a-b**) and *mot2*Δ (**c-d**) $REIII_{mut}$ cells grown at 32°C. A density bar represents the fraction of the most dense bin. Panels correspond to the second and third isolates for both backgrounds. **e** RT-qPCR analyses of *cenH* transcripts (mean±SD; n=3; normalized to *act1+*; relative to wt) in cells of the indicated genetic backgrounds. Two-tailed Student's *t*-tests were used to calculate *p*-values. Individual data points are represented by black circles.

Supplementary Fig. 4. Caf1 and Mot2 impact heterochromatin spreading at subtelomeres but not centromeres. a-b) H3K9me3 ChIP-qPCR analyses (% input; mean±SD; n=3) in cells of the indicated genetic backgrounds. Immunoprecipitations without antibodies (no Ab) were performed to determine background levels. **c)** RT-qPCR analyses of subtelomeric transcripts (mean±SD; n=3; normalized to *act1+*; relative to wt) in cells of the indicated genetic backgrounds. **a-c)** Numbers correspond to the different primer pairs used in qPCR reactions and whose localization is indicated on the scheme below each graph. Two-tailed Student's *t*-tests were used to calculate *p*-values. Individual data points are represented by black circles. In the scheme in **a)**, vertical black lines in the imr1R region represent tRNA genes that delimit heterochromatin boundaries, beyond which H3K9me3 is not enriched.

Supplementary Fig. 5 (related to Fig. 4). Importance of Caf1 and Mot2 catalytic activities in gene silencing and heterochromatin spreading. a), d) Western blots showing total 2xFLAG-tagged wild type or mutant Caf1 (**a**) and Mot2 (**d**) expressed from the pREP41 vector. Anti-CDC2 antibody was used as loading control. **b)** 5-fold serial diluted silencing assay using the *ura4+* reporter gene inserted at the mating type locus (mat3M::*ura4+*). Cells of the indicated

genotypes were plated on both non-selective (EMM-LEU) and 5FOA-containing (EMM-LEU+5FOA) media. **c)** RT-qPCR analyses of *ura4+* transcripts (mean±SD; n=4; normalized to *act1+*; relative to wt pREP41) in cells of the indicated genetic backgrounds. Two-tailed Student's *t*-tests were used to calculate *p*-values. Individual data points are represented by black circles.

Supplementary Fig. 6 (related to Fig. 5). The anti-silencing factor Epe1 opposes Ccr4-Not in gene silencing and heterochromatin spreading. a) Northern blot showing *ura4+* mRNA levels from total RNA samples in the indicated genetic backgrounds (mat3M::*ura4+*). The PCR probe overlapping the 3' end of *ura4+* also detects the endogenous *ura4-DS/E* mini-gene. BET-stained ribosomal RNAs serve as a loading control. **b)** Western blot showing total TAP-tagged Epe1 in the indicated genetic backgrounds. Anti-CDC2 antibody was used as loading control. **c-f)** Two-dimensional-density squarebin plots showing the red-normalized green and orange fluorescence for *caf1Δ epe1Δ* (**c-d**) and *mot2*Δ *epe1Δ* (**e-f**) $REIII_{mut}$ cells grown at 32°C. A density bar represents the fraction of the most dense bin. Panels correspond to the second and third isolates for both backgrounds.

Supplementary Fig. 7. Mutation of the Epe1 jumonji domain suppresses silencing defects in *caf1*Δ and *mot2*Δ cells. a) Domain organization of the Epe1 protein. **b)** 5-fold serial diluted silencing assay using the *ura4+* reporter gene inserted at the mating type locus (mat3M::*ura4+*). Cells of the indicated genotypes were plated on both non-selective (YES) and 5FOA-containing (YES+5FOA) media. **c)** RT-qPCR analyses of *ura4+* transcripts (mean±SD; n=4; normalized to *act1+*; relative to wt) in cells of the indicated genetic backgrounds. **d)** Western blot showing total wild type or H297A TAP-tagged Epe1 in the indicated genetic backgrounds. Anti-CDC2 antibody was used as loading control. **e)** ChIP-qPCR analyses (% input; mean±SD; n=4) of the

indicated strains in the mat3M::*ura4*+ background. Immunoprecipitations without rabbit IgG (no Ab) were performed to determine background levels. **c)**, **e)** Two-tailed Student's *t*-tests were used to calculate *p*-values. Individual data points are represented by black circles.

Supplementary Table 1. *S. pombe* strains used in this study.

Supplementary Table 2. Plasmids used in this study.

Supplementary Table 3. Oligonucleotides used in this study.
