# Supplementary figures and images for: A dual, catalytic role for the fission yeast Ccr4-Not complex in gene silencing and heterochromatin spreading
Source: Genetics. 2023 Jun 6;224(4):iyad108. doi: 10.1093/genetics/iyad108 (PMC10411572; doi:10.1093/genetics/iyad108)

**(a)**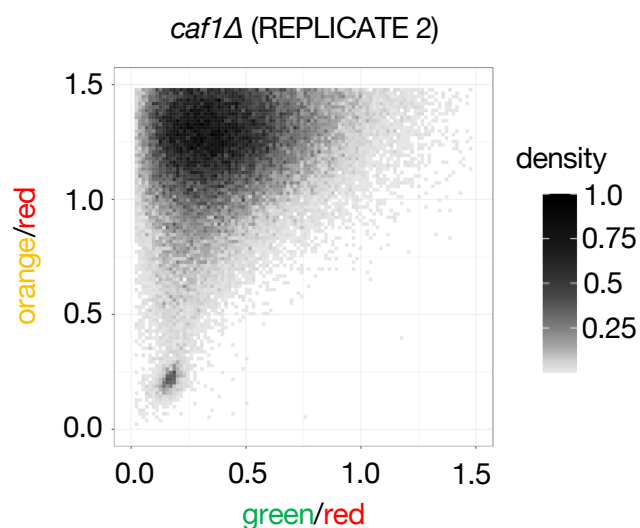**(b)**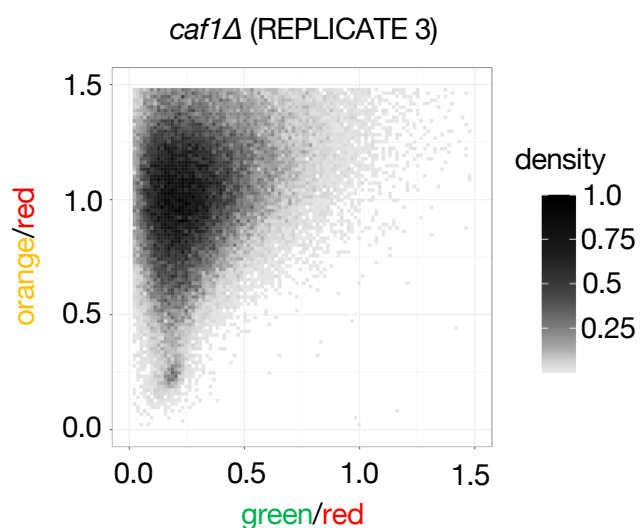**(c)**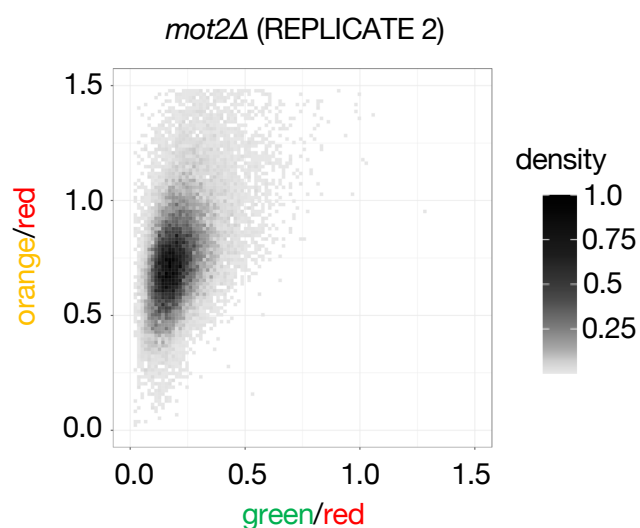**(d)**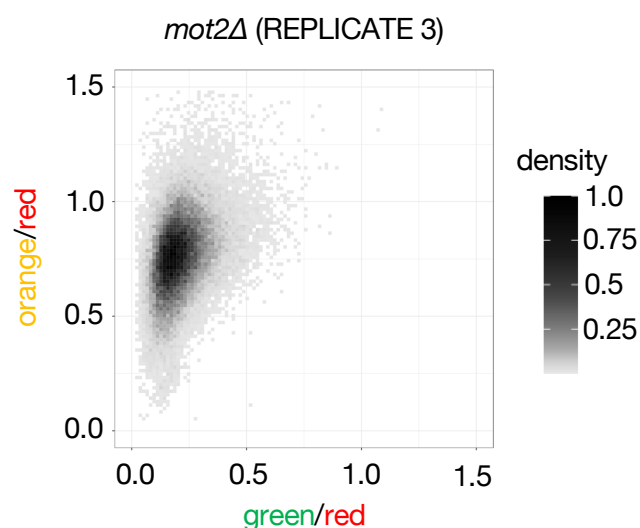**(e)**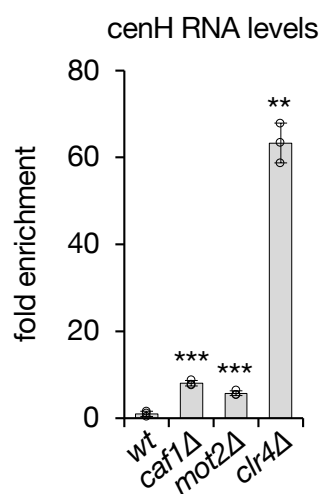

**Supplementary Fig. 3. The Ccr4-Not subunits Caf1 and Mot2 regulate heterochromatin spreading.**

Supplement: iyad108_Supplementary_Data [file iyad108_supplementary_data.zip › Supplementary_Figure_3_GENETICS-2023-306219.pdf]
